# What Is the Community Pharmacists’ Role in Supporting Older Australians with Palliative Care Needs?

**DOI:** 10.3390/healthcare9050489

**Published:** 2021-04-21

**Authors:** Paul Tait, Amal Chakraborty, Kelly Jones, Jennifer Tieman

**Affiliations:** 1 Southern Adelaide Palliative Services, Flinders Medical Centre, SA Health, Bedford Park, SA 5042, Australia; 2 Research Centre for Palliative Care, Death and Dying, College of Nursing and Health Sciences, Flinders University, Bedford Park, SA 5042, Australia; amal.chakraborty@flinders.edu.au (A.C.); kelly.jones@flinders.edu.au (K.J.); jennifer.tieman@flinders.edu.au (J.T.)

**Keywords:** aged, caregivers, community pharmacy services, death, delivery of health care, general practice, medication therapy management, palliative care, pharmacists, surveys and questionnaires

## Abstract

As the population ages, the number of older populations globally requiring palliative care is rapidly growing, requiring services of multidisciplinary teams—including community pharmacists. The aim of this study is to describe the community pharmacists’ perceived role in providing services to community dwelling older Australians receiving palliative care. Utilising an eight-domain End of Life Directions for Aged Care (ELDAC) care model, a national cross-sectional questionnaire was designed and undertaken online with Australian community pharmacists. Respondents were asked questions relating to socio-demographic characteristics, practice characteristics, and scope of services provided. Of the 62 pharmacists who responded to the questionnaire, 51 were included in the final data analysis and reporting. Pharmacists working in dispensing roles made up about half of the respondents, while the remainder worked in settings such as general practice, residential aged care, or providing medication review services. Pharmacists can identify patients with indicators of poor life expectancy and mostly work with older Australians daily. Dispensing and non-dispensing pharmacists offer a range of services that complement each other. Organisations caring for the aged should consider the role of the pharmacist, in caring for people with palliative care needs, along with their carers.

## 1. Introduction

As the population increases, and ages, the number of older Australians with palliative care needs is growing [1]. Varying levels of complexity in health and social needs, which may change over time, requires palliative care to be provided by a variety of health disciplines across both the acute and primary care settings [2].

Palliative care is described as active and multidisciplinary care for people living with a life-limiting illness, no longer responding to treatment [2]. Traditionally, palliative care has been a specialty role. With the push for people to receive care in their own homes, people who are in the last phase of their life have become “everybody’s business”: everybody across the health and aged care workforce has a role [2]. This includes clinicians working across a range of non-government organisations (NGOs), such as general practices, Aboriginal health services, and allied health and community pharmacy services. A range of government funding levers apply to many of these services (e.g., dispensing of prescriptions, pharmacist led medication reviews, and general practitioner (GP) appointments) leaving minimal out-of-pocket expenses for the consumer. In addition to these arrangements, older Australians can also apply for federally funded aged care packages, which are intended for those with more complex care needs. Once allocated, an aged care package is administered by federal government approved aged care providers. Depending on the level of package, older Australians can use these to obtain services to remain independent at home (through the receipt of home care (HC)), access respite or transitional care, or to access permanent accommodation in a residential aged care home (in receipt of residential aged care (RAC)) [3]. As such, there is significant complexity in how palliative care is delivered in Australia.

Older Australians in HC and RAC settings have an increased risk of medication misadventure [4,5,6,7,8,9,10]. Furthermore, people with palliative care needs have additional complexities in how their medicines are managed, resulting from, for example, poor swallowing, the need for subcutaneous administration of medications, and a greater reliance on the carer for medication management [11,12]. The pharmacist forms an important part of the multidisciplinary care system for older people coming to the end of their life in both HC and RAC settings. The federal government too understands the impact that medication misadventure has on wellbeing of our community. In 2019, they added quality use of medicines (QUM) and medicines safety as a tenth Australian health priority area; the importance of safe and quality use of medicines in Australia is underpinned in policy [13]. 

Pharmacists are experts in medicines and as the Australian health system evolves, their roles are becoming increasingly diverse [14]. In Australia, pharmacists are funded to perform a range of tasks, including supplying medicines, patient-level activities (e.g., medication review), clinical governance (participation in accreditation programs), and education and training [14]. While pharmacists are traditionally based in community pharmacies (dispensing pharmacists), the literature describes the role of the pharmacist working remotely from the community pharmacy (non-dispensing pharmacists) [15,16,17]. Importantly, non-dispensing pharmacists require accreditation to provide federal government funded medication reviews. Roles may be embedded in an Aboriginal health service, RAC home, or general practice. In addition, non-dispensing pharmacists may work independently, providing medication reviews, in the person’s own home [14,15,16,17]. 

End of Life Directions for Aged Care (ELDAC) is a project funded by the Federal Government Department of Health; it develops a range of services and resources to support clinicians working across NGOs to deliver quality palliative care for older Australians, defined hereon as life expectancy to be less than 12 months [18]. These are built upon the ELDAC care model (see Table 1), which describes eight key care domains that have been fundamental to Australian best practice in providing care in the last phase of life [19]. Understanding the role of dispensing and non-dispensing pharmacists and the services they provide, in the context of these domains, may enable more empirical use of relevant and effective resources or approaches for pharmacists providing palliative care in the community. In 2019, ELDAC conducted a rapid review describing the roles that pharmacists undertake in the community. The review of the international published literature failed to identify a single paper that examined the broader role of the pharmacist with older people with palliative care needs and their carers throughout the entire palliative care journey, into bereavement [7]. 

The aim of this study was to describe the community pharmacists’ perceived role in providing services to community dwelling older Australians receiving palliative care.

## 2. Materials and Methods

### 2.1. Design and Sampling

This study utilised a cross-sectional, convenience sample of Australian registered pharmacists. The eight-domain ELDAC care model [19] was used as the conceptual framework to capture dimensions of palliative care in development of the community pharmacy questionnaire (the questionnaire). The electronic questionnaire was composed of three distinct sections:

- Section 1: Socio-demographic and role characteristics of the respondents;
- Section 2: Practice characteristics;
- Section 3: Scope of services provided.

Pharmacists with a dispensing role were asked six extra questions to identify some context to their work around the dispensing of medicines. A participant information sheet (PIS) was developed to explain the research project to potential respondents and request their involvement. The questionnaire and PIS were reviewed and trialled with pharmacist and non-pharmacist peers for readability and comprehension. The questionnaire was delivered in the Checkbox Survey system [20].

The Australian registration body for pharmacists was approached to distribute the questionnaire. They declined, explaining they do not use their database to gather workforce information for external agencies. As such, several professional organisations, relevant to pharmacy practice, were approached to distribute the questionnaire, through their digital channels, including: the Pharmaceutical Society of Australia (PSA), organisations supporting pharmacist education, several palliative care networks, and primary health networks. Digital channels included e-newsletters and social media posts conducted via Twitter, Facebook, and LinkedIn. Organisations were encouraged to send several reminder social media and e-newsletter postings, which were made during the questionnaire period to prompt potential respondents to complete the survey. Self-selection and snowball sampling, using these networks as seeding opportunities, were used.

Pharmacists practicing in dispensing and non-dispensing roles with community dwelling older people were eligible to complete the questionnaire. Pharmacists were excluded if they worked solely in a hospital setting, were not currently in the workforce, or did not offer services to older Australians (e.g., children’s immunisation service).

Calculation of sample size was performed using StatCalc software (version 9). An estimated sample size of 62 was required to detect at least an 80% proportion of participants who care for community dwelling older adults at a significance criterion of 0.05 and a power of 80. A response rate of 82% was achieved.

### 2.2. Ethical Consideration

Full approval to conduct this study was gained from the Human Research Ethics Committee of the Flinders University (Project Ref # 8517).

### 2.3. Data Collection

The analysis includes data collected between 18 March 2020–21 November 2020. participation and submission of the online questionnaire implied informed consent. 

### 2.4. Data Items

Data were arranged and described according to socio-demographic and role characteristics (see Table 2), practice characteristics (see Table 3), and scope of services provided (see Table 4). Table 5 and Table 6 provide more description to some of the questions. Responses received were from a combination of closed and open-ended questions. The questionnaire was designed to take approximately 20 min to complete. No mandatory questions were imposed, and respondents were not identifiable in any of the responses received.

### 2.5. Data Analysis

Data extracted from the Checkbox Survey system platform were directly exported to and analysed using SPSS software, version 25.0 [21]. Descriptive statistics were used to analyse data. At each analysis point, quantitative measurements were summarised using frequency and percentages for categorical variables. 

## 3. Results

A total of 3821 questionnaire impressions were obtained, indicating that it was opened by a recipient of the electronic link. Of those who opened the questionnaire, 62 progressed past the welcome page, which described the aims of the study and linked to the PIS. Eleven respondents were excluded from the data analysis as they did not meet the inclusion criteria of currently working in a clinical context in a community setting. Results are provided as a proportion of respondents answering the individual question.

### 3.1. Respondents

In total, 51 respondents completed the questionnaire and were included for analysis and reporting.

#### 3.1.1. Socio-Demographic and Role Characteristics of the Respondents

Table 2 shows the demographic characteristics of all respondents. Respondents were predominantly female (76.5%) and were aged less than 40 years (60.0%), which corresponds with national registration data (AHPRA and the National Boards (2020). AHPRA and National Boards annual report 2019/20. Melbourne, Australian Health Practitioner Regulation Agency). Most had no formal postgraduate qualifications (80.4%), while about half (54.0%) of the respondents had practiced pharmacy for more than 10 years. Most (59.1%) of the respondents worked full-time. Pharmacists working in dispensing roles (52.9%) in a community pharmacy setting made up about half of the respondents, with the remainder working in clinical roles, such as working in general practice (see Table 2).

Many (45.5%) of the respondents were accredited to provide federal government funded medication reviews.

#### 3.1.2. Practice Characteristics

Practice characteristics are provided in Table 3. Most respondents (85.4%) provided daily services to older Australians, in their capacity as a pharmacist. Respondents were aware of patients receiving a median of 6 different types of federal government funded aged care services. The most likely aged care services for pharmacists to be aware of included: patients living in a residential aged care homes (87.5%), respite care (85.0%), home care package (76.9%), after-hospital transition care (75.0%), Commonwealth Home Support Program (72.5%), and short-term restorative care (67.5%). 

Some population groups have been identified by the Australian government as having specific needs in the healthcare sector. Of the ten specific needs populations listed, respondents identified a median of eight groups using their services. The least likely groups for a pharmacist to be aware of were care leavers (42.5%) and the homeless (40.0%).

Most respondents (85.3%) reported that they were aware of people with palliative care needs using their services, with information from another healthcare provider (92.0%) or the carer (91.7%) being the most likely means they learnt this information. A little less than half of the respondents (41.4%) estimated that 25% or less of their older clients are likely to die in the next 12 months.

Respondents were asked if they were aware of patients using services in the last 12 months who were experiencing six specific care needs associated with death in the coming months. Most respondents (65.0%) identified patients with all six specific care needs listed, including: persistent and troublesome symptoms (100.0%), people living in a residential aged care home or needing care at home (100.0%), and two or more unplanned hospital admissions (97.5%).

#### 3.1.3. Scope of Services Provided

Of the eight elements of the ELDAC care model, respondents recognised a median of 4 of these elements in their daily practice. These were working together (88.2%), providing palliative care (58.8%), assess palliative care needs (55.9%), respond to deterioration (50.0%), and manage dying (50.0%). The least recognised element of the ELDAC care model was advance care planning (32.4%).

Table 4 describes the services that dispensing and non-dispensing pharmacists provide.

Only one sixth of the respondents had witnessed or countersigned an advance care plan (17.6%), while less than half (44.1%) had provided information on advance care planning to patients and carers. More dispensing pharmacists had provided information on ACP, while more non-dispensing pharmacists had witnessed or countersigned an Advance Care Directive.

Most respondents (82.4%) recognised that they had recommended medicines be deprescribed.

Dispensing and clinical software can provide prompts to the user about care needs. Pharmacy software used to dispense prescriptions or provide clinical care were mostly either Fred Dispense^®^ (31.3%) or Minfos^®^ (15.6%).

Freely available resources can be used to provide evidenced based information to the people that use their services. Respondents were asked if they had used or were unaware of several freely available palliative care resources. Table 5 groups the resources into resource types. Of the resources listed, pharmacists mostly used the clinical tools. These included the opioid calculator app (62.5%), the opioid calculator website (56.3%), and the palliMEDS smartphone application (37.5%). Respondents were least likely to be aware of experiential training programs that were available (e.g., The Program of Experience in the Palliative Approach (PEPA)) in Australia. Evidence based resources (e.g., ELDAC and CareSearch) were the least used resources listed (see Table 5).

Most respondents (68.7%) described the involvement of carers always or often, when providing advice, education, and/or resources relating to managing an individual’s medicines.

Several issues pertaining to the way an individual manages medicines or risk of a medication misadventure (from previous adverse drug reaction to mobility issues) were listed in the questionnaire. In addition, there were questions pertaining to indicators for approaching the end of life (see Table 6). Respondents were asked if they documented these. The most likely issues documented were previous adverse drug reactions (90.3%), presence of poly pharmacy (74.1%), swallowing difficulties (67.7%), and poor eyesight (64.5%). Pharmacists were least likely to document mobility concerns (58.1%), poor health literacy (*n* = 17/31, 54.8%), poor liver function (51.6%), or presence of heart failure (51.6%). 

When communicating with GPs and nurse practitioners about patient specific information, pharmacists used a multitude of ways, including: facsimile (100.0%), telephone (100.0%), email (93.3%), electronic or written health record (73.3%), face-to-face (60.0%), and letter (53.3%). Less than one in five respondents used secure messaging services to communicate.

Two thirds (64.5%) of the respondents had never participated in a case conference of a patient with palliative care needs.

Eighty percent of respondents said they could recall bereaved clients discussing their loss with them. Sixty percent of the respondents were concerned enough for the bereaved individual to recommend psychological support. Dispensing pharmacists were more likely than non-dispensing pharmacists to be involved with these activities.

**Dispensing Pharmacists (subgroup)**

All respondents working in a dispensary offered home delivery services, provided a dose administration aid service, were involved in the return of unwanted medicines (RUM) programme, and made available a staged supply service. Two thirds of respondents (66.7%) worked in pharmacies with extended hours.

Community pharmacies can offer a range of supportive equipment either through hiring out or selling these items. The focus of products in stock tended to be ambulant mobility aids and accessories (86.7%) as well as bathing, toileting, and continence aids (66.7%). Pharmacists were more likely to advocate for the patient by calling another pharmacy to see if they had stock or to order stock in upon request if the product was unavailable.

Respondents offered a range of federal government funded clinical services from the pharmacy. Most pharmacies offered a free in pharmacy medicines management review, called a MedsCheck^®^ (93.3%). This can be recommended by any health care providers (HCP), the carer, or the individual living with a life-limiting illness. Further, most of the pharmacies offered a federal government funded medication review service, a more comprehensive service performed by accredited pharmacists. 

The National Palliative Care Symptom Management Medicines (NPCSMM) list is an Australian list of ten commonly prescribed formulations (mainly for subcutaneous administration) useful in the management of symptoms in the last days of life. Six out of 14 respondents (42.9%) knew of the NPCSMM list. 

If pharmacists received a prescription for a formulation that they did not stock, nearly half (46.7%) would advocate for their patient by calling another pharmacy in the area to borrow stock. Only a fraction of respondents would contact the prescriber to discuss alternative arrangements.

About half of the respondents (53.3%) reported that they have arranged to carry formulations based on discussions with prescribers in their area. All said they were keen to continue carrying this stock based on the following:

- Having a formal relationship with an RAC Home or a hospice;
- Working in areas with a high proportion of older people;
- Overcoming delays in accessing stock due to rural location;
- Providing a public service—knowing that carrying these medicines reduces the stress for the individual, their carer, and the prescriber;
- Responding to prescribing patterns in the area.

## 4. Discussion

Pharmacists in both dispensing and non-dispensing roles offer a broad range of services to older Australians receiving either HC or RAC. These data describe how both groups play important and complimentary roles in the care of older Australians living with palliative needs. Most of the respondents had older people accessing their services daily, making the pharmacist an important part of the multidisciplinary team.

Advance care planning is a way to think ahead, to describe what is important to an individual, ensuring other people know their wishes for the future. It includes conversations and documentation of wishes. The poor engagement of the pharmacist in this space may be reflective of the workplace setting, lack of opportunities to participate, or lack of insight of the pharmacist’s role in advance care planning. Conversations as part of a medication review could aim to broach issues, such as goals of care.

Proactively, HCPs need to consider whether an individual could have changes indicating that death is foreseeable. Recognising that they have palliative care needs can impact a range of issues, including the goals of medication reviews, support of caregivers in managing medicines, and putting in place the services to make management of medicines easier with reduced function. These data show that pharmacists were able to identify specific care needs associated with approaching the end of life as well as indicators for predicting medication misadventure. In addition, pharmacists were able to identify people with palliative care needs. The poor response rate limits any discussion of and association with identifying these factors and the services they offered by the pharmacist. Pharmacy software tools may be useful in this regard to flag specific groups of patients with palliative care needs for consideration. Some flags, such as age (e.g., older than 65 years), may be straightforward to incorporate into pharmacy software. Others, such as multiple unplanned admissions to hospital, may require multiple datasets to communicate with each other or be required information to be included by medical offers when referring for pharmacy services. Federally funded levers, such as medication reviews, may be useful ways to engage the pharmacist early in care as well as to share information that the pharmacist may be unaware of and support their role in care provision. A recent Australian study reported that few individuals receive a timely medication review upon entering permanent RAC; this is despite clear guidelines recommending this [24]. At present, there is no clear guidance recommending older Australians receiving formalised HC to be provided with a medication review. While this is a small sample, it indicates opportunities for medication reviews to be integrated into standard care of all people receiving federally funded aged care packages for support in their own home. 

Having a carer is a significant enabler for someone with palliative care needs to receive HC [25]. Over the last decade, the literature clearly highlights the support that carers need to assist them in providing care in a manner that also considers their own wellbeing [26,27,28]. The results described how two thirds of respondents are likely to involve carers when providing advice, education, and/or resources relating to managing an individual’s medicines. Pharmacists could be better engaged with carers. This is imperative, when the relationship between HCPs and carers can become complex, particularly at the end of life, when the risk of medication misadventure is high [25]. While pharmacists are not trained therapists, they can refer people, at risk of issues pertaining to their grief, back to their GP. Interestingly, dispensing pharmacists were more likely to have conversations about bereavement and to refer for support. This can be explained by the ongoing relationship they have with carers through the ongoing dispensing of medicines.

Pharmacists tend to work remotely to the healthcare team and can often be unable to anticipate care needs, such as which medicines to stock [29]. Case conferences have been shown to improve communication; improve coordination of care; and clarify goals of care and support for patient, families, and carers [30]. The poor response of pharmacist involvement in case conferences may be reflective of medicines management not being considered, as the case conference is being arranged. With efforts to embed Australian pharmacists in RAC and general practice underway, improved communication with the pharmacist is foreseeable [9,31]. The recent COVID-19 pandemic has resulted in the adoption of innovative telehealth approaches that could contribute to improved communication relating to medication management [32,33]. In Australia, the My Health Record (MHR), facilitated by the federal government, is a secure online summary of an individual’s health information. It is available to all Australians. Healthcare providers authorised by their healthcare organisation can access it to view and add patient health information, including dispensing information. The MHR is limited in that pharmacists in both hospital and community settings are unable to upload findings from medication reviews. Improving the MHR to allow for pharmacists to contribute more than simply dispensing data would assist in this information being available for all prescribers and pharmacists caring for the individual.

Medicines play an important role in supporting symptom control. In the last days of life (terminal phase), there is also a loss of ability to swallow leading to the medicines being administered subcutaneously or as oral liquids. We identified an association between pharmacists stocking injectable medicines and knowing about the NPCSMM list. This indicates that promotion of the NPCSMM list has resulted in some changes in practice. The finding that most of the respondents carried many of the formulations in the NPCSMM list is contrary to previous studies [29]. This could be explained with variability in this small dataset. 

Organisations caring for the aged should consider the role of the pharmacist in caring for people with palliative care needs, along with their carers.

## 5. Limitations

The questionnaire generated over 3800 impressions, which was not reflected in the final number completed. This could be due to pharmacists not having enough time nor interest in completing it. The questionnaire was distributed within the COVID-19 pandemic, which may have limited the response rate. It was challenging that the national regulation body for pharmacists was unable to distribute the questionnaire. While numbers of respondents provided some indication of the role of pharmacists in the care of people with palliative care needs, more work needs to be invested into understanding their needs.

## 6. Conclusions

The role of the pharmacist in Australia is diverse. Building on clinical expertise, several innovative pharmacist roles have developed in recent years. This paper describes how pharmacists work with older people daily. They provide a variety of services for people with palliative needs as well as their carers. Engaging with the pharmacist early in the palliative trajectory of the patient is important. Organisations must develop systems that embed pharmacists or pharmacy services in usual patient care processes. 

## Figures and Tables

**Table 1:** The eight domains of the End of Life Directions for Aged Care (ELDAC) care model [19].

Domain No.	Domain Name	Domain Description
1	Advance Care Planning	Documenting how a person would prefer (including wishes) to be cared for at the end of life
2	Recognise End of Life	Proactively considering whether the person could have changes indicating that death is foreseeable
3	Assess Palliative Care Needs	Comprehensive identification and planning of palliative care and addressing a person’s needs at the end of life
4	Provide Palliative Care	Delivering palliative care, reassessing needs, and monitoring for changes
5	Work Together	Working with a team of multidisciplinary professionals and services to provide coordinated palliative care
6	Respond to Deterioration	Identifying changing needs, caring for a person who is quickly approaching death, and updating care plans
7	Manage Dying	Having an appropriate plan in place to manage the last days of life
8	Bereavement	Supporting family, friends, residents, and staff with grief and loss after a death

**Table 2:** Socio-demographic and role characteristics of the respondents.

Characteristics: (N)		All Respondents	
		*n*	%
Gender: (51)	Female	39	76.5
	Male	12	23.5
Age: (50)	<40 years	30	60.0
	>40 years	20	40.0
Postgraduate qualification: (51)	No	41	80.4
	Yes	10	19.6
Experience as pharmacists: (50)	10 years or less	23	46.0
	>10 years	27	54.0
Employment status: (44)	Part-time-Casual	18	40.9
	Full-time	26	59.1
Work in a dispensing role: (51)	Yes	27	52.9
	No	24	47.1
Accredited to provide Commonwealth funded medication reviews: (44)	Yes	20	45.5
	No	24	54.5

**Table 3:** Practice characteristics.

Characteristics: (N)		All Respondents	
		*n*	%
Do you provide daily services for older Australians? (41)	Yes	35	85.4
	No	6	14.6
How many specific population groups are you aware of using your services (ten examples offered)? (40)	0–5	17	33.3
	6–10	34	66.6
Are you aware of older Australians receiving at least one Federal Government funded aged care service? (40)	Yes	39	97.5
	No	1	2.5
In your opinion, will at least 25% of the older Australians using your service die in next 12 months? (41)	Yes	18	44.0
	No	23	56.0
Are you aware of any people using your services in the previous 12 months experiencing at least 4 indicators of clinical deterioration (six examples offered)? (40)	Yes	39	97.5
	No	1	2.5
Are you aware of people with palliative care needs using your services? (34)	Yes	29	85.3
	No	5	14.7

**Table 4:** Scope of services provided by dispensing and non-dispensing pharmacists.

Characteristics: (N)		Respondents in Dispensing Role		Respondents in Non-Dispensing Role		All Respondents	
		*n*	%	*n*	%	*n*	%
Do you provide ACP information to patients and their carers? (34)	Yes	11	32.4	4	11.8	15	44.1
	No	6	17.6	13	38.2	19	55.9
Have you witnessed or countersigned an Advance Care Directive? (34)	Yes	2	5.9	4	11.8	6	17.6
	No	15	44.1	13	38.2	28	82.4
Have you recommended, to a prescriber, that a medicine be deprescribed? (34)	Yes	13	38.2	15	44.1	28	82.4
	No	4	11.8	2	5.9	6	17.6
Have you involved the individual’s carer when providing advice, education and/or resources for older Australians, most of the time (i.e., often or always)? (32)	Yes	12	37.5	10	31.3	22	68.7
	No	4	12.5	6	18.8	10	31.3
What is the main pharmacy software program used in practice? (32)	Fred dispense	7	21.9	3	9.4	10	31.3
	Minfos	4	12.5	1	3.1	5	15.6
	Z software	3	9.4	1	3.1	4	12.5
	Other	1	3.1	5	15.6	6	18.7
	None	1	3.1	6	18.8	7	21.9
Have you ever participated in a case conference? (31)	Yes	6	19.4	5	16.1	11	35.5
	No	10	32.3	10	32.3	20	64.5
Have bereaved clients discussed their loss with you? (31)	Yes	16	51.6	9	29.0	25	80.6
	No	0	0.0	6	19.4	6	19.4
Have you ever referred a bereaved client for psychological support? (31)	Yes	15	48.4	4	12.9	19	61.3
	No	1	3.2	11	35.5	12	38.7
**Dispensing Pharmacist Specific Questions**							
What range of supportive equipment is hired or sold by the pharmacy? (15)	0–4	9	60.0	n.a.	n.a.	9	60.0
	5–8	6	40.0	n.a.	n.a.	6	40.0
Which medication review services are offered by the pharmacy you work in? (15)	MedsCheck	14	93.3	n.a.	n.a.	14	93.3
	Medication review Services offered by an accredited pharmacist	12	80.0	n.a.	n.a.	12	80.0
How many ways do you communicate with GPs or nurse practitioners about patient specific information (eight examples offered)? (15)	0–4 ways	4	26.7	n.a.	n.a.	4	26.7
	5–8 ways	11	73.3	n.a.	n.a.	11	73.3
How Many medicines from the “Palliative Care Symptom Management Medicines List for Australians living in the community” are stocked by the pharmacy? (15)	0–5 medicines	5	33.3	n.a.	n.a.	5	33.3
	6–10 medicines	10	66.7	n.a.	n.a.	10	66.7
Are you aware of the National “Palliative Care Symptom Management Medicines” list? (14)	Yes	6	42.9	n.a.	n.a.	6	42.9
	No	8	57.1	n.a.	n.a.	8	57.1
Does the pharmacy have an arrangement to stock a range of subcutaneous medicines based on discussions with prescribers in the area? (15)	Yes	8	53.3	n.a.	n.a.	8	53.3
	No or unsure	7	46.7	n.a.	n.a.	7	46.7

n.a. = not applicable.

**Table 5:** Awareness of evidence based palliative care dosing and information resources.

Free Evidence-Based Palliative Care Resources	Yes		No		Unaware	
	*n*	%	*n*	%	*n*	%
Clinical Tools	19	59.4	11	34.4	2	6.3
Experiential Training Programs	8	25	14	43.8	10	31.3
Evidence Based Resources	7	21.9	17	53.1	8	25.0
Advance Care Planning Resources	11	34.4	15	46.9	6	18.8

**Table 6:** Pharmacists’ awareness of care needs for patients approaching end-of-life and indicators for predicting medication misadventure.

Awareness of Care Needs for Patients Approaching End-Of-Life (*n* = 40) [22] *			Systems to Identify Patients for Predicting Medication Misadventure (*n* = 31) [23]		
Indicators	*n*	%	Indicators	*n*	%
Persistent, troublesome symptoms	40	100.0	Previous adverse drug reaction	23	74.2
Lives in a residential aged care home or needs care at home	40	100.0	Polypharmacy (more than 5 medicines)	21	67.7
Two or more unplanned hospital admissions	39	97.5	Multimorbidity	16	51.6
Needs help with personal care	38	95.0	Swallowing difficulty	14	45.2
Requests supportive and palliative care or treatment withdrawal	33	82.5	Poor eyesight	13	41.9
Weight loss (5–10%) and/or body mass index <20	30	75.0	Poor renal function	13	41.9
			English as a second language	12	38.7
			Poor hearing	12	38.7
			Cognitive decline	11	35.5
			Heart failure	11	35.5
			Poor liver function	11	35.5
			Poor mobility	11	35.5
			Poor dexterity or poor fine manipulative skills	10	32.3
			Poor health literacy	9	29.0

* Supportive and Palliative Care Indicators Tool (SPICTTM) requires identifying two or more general indicators of deteriorating health as a prompt for someone approaching the end of their life [22].

## Data Availability

Data on this study may be made available upon reasonable request to the authors: paul.tait@flinders.edu.au; jennifer.tieman@flinders.edu.au.
